# High Interferon-γ Uniquely in Vδ1 T Cells Correlates with Markers of Inflammation and Axonal Damage in Early Multiple Sclerosis

**DOI:** 10.3389/fimmu.2017.00260

**Published:** 2017-03-09

**Authors:** Avadhesh Kumar Singh, Lenka Novakova, Markus Axelsson, Clas Malmeström, Henrik Zetterberg, Jan Lycke, Susanna L. Cardell

**Affiliations:** ^1^Department of Microbiology and Immunology, Institute of Biomedicine, Sahlgrenska Academy, University of Gothenburg, Gothenburg, Sweden; ^2^Department of Clinical Neuroscience, Institute of Neuroscience and Physiology, Sahlgrenska Academy, University of Gothenburg, Gothenburg, Sweden; ^3^Department of Psychiatry and Neurochemistry, Institute of Neuroscience and Physiology, Sahlgrenska Academy, University of Gothenburg, Mölndal, Sweden; ^4^Clinical Neurochemistry Laboratory, Sahlgrenska University Hospital, Mölndal, Sweden; ^5^Department of Molecular Neuroscience, UCL Institute of Neurology, London, UK

**Keywords:** cerebrospinal fluid, gamma-delta T cells, interferon-gamma, multiple sclerosis, natalizumab, Vdelta1 T cells

## Abstract

We have identified a population of T lymphocytes in peripheral blood, Vδ1 TCRγδ T lymphocytes, which unexpectedly was uniquely expressing high production of interferon-γ in newly diagnosed, untreated multiple sclerosis (MS) patients. IFN-γ production in this population distinctly correlated to parameters of clinical disease activity, inflammation, and neuronal damage. These Vδ1 T lymphocytes belong to a population of innate T lymphocytes that recognize antigen in the context of CD1d/CD1c and which include reactivity to the myelin glycosphingolipid sulfatide. Importantly, patients treated with natalizumab, blocking leukocyte transmigration to central nervous system, had completely normalized levels of interferon-γ-producing Vδ1 T lymphocytes. A biomarker and early sign of demyelinating disease in MS is much warranted and would help identify immunopathogenesis and prognosis of disease as well as monitor success with adequate treatment. The present study identifies the Vδ1 T lymphocytes as an early marker of MS and a possible link to understanding the disease etiology.

## Introduction

Multiple sclerosis (MS) is a chronic inflammatory disease of the central nervous system (CNS) where the myelin sheath around nerve fibers becomes the target of an autoimmune attack. This leads to demyelination, axonal loss, and subsequent progressive neurologic functional deficits (1). In a majority of patients, the initial phase of the disease is characterized by relapsing–remitting MS (RRMS), periods of exacerbation of neurological symptoms followed by substantial remission that can last for months and sometimes several years. The underlying mechanisms involved in the initiation of disease and the processes that are responsible for the progression to chronic autoimmunity are poorly understood. Although geographical distribution, lifestyle, and environmental factors may be contributing in MS, extensive investigations including genetic association studies in animal models and MS patients support a primary role for cell-mediated immune mechanisms in the disease pathogenesis (1).

A pathogenic role for CD4^+^ Th cells is well established in MS, but the specific contribution of different Th subsets to the disease process has remained elusive. Early studies indicated a primary pathogenic role for IFN-γ-producing Th1 cells, however, this view has been challenged in recent years when IL-17-producing Th17 cells have been viewed as the main actors in the course of MS. The current notion is that a mix of Th1/Th17 cells and associated proinflammatory cytokines act in concert to precipitate disease, and that the production of combinations of IL-17, IFN-γ, and granulocyte-macrophage colony-stimulating factor characterize pathogenic Th cells (2–7). By contrast, IL-10-producing Th cells are considered to be protective.

γδ cells express a distinct TCR composed of the TCR γ- and δ-chains (8). They represent a group of cells called innate-like lymphocytes characterized by rapid activation and early involvement during immune responses. γδ cells are suggested to play a role in both MS and the murine model for the disease, experimental autoimmune encephalomyelitis (EAE) (9–11). Clonally expanded γδ cells have been found accumulated in acute, demyelinating MS brain lesions (12) and in cerebrospinal fluid (CSF) from recent-onset MS patients (13), strongly indicating that γδ cells are involved in MS pathogenesis. γδ cells are particularly abundant in peripheral tissues, most notably, the skin and intestinal epithelium where they monitor early signs of tissue infection and stress. Human γδ cells are dominated by two main populations defined by their expression of the Vδ1 or Vδ2 segments of the TCR δ-chain. The Vδ1 and Vδ2 T cells predominate in the mucosal epithelium and circulation, respectively.

In general, the immunobiology of Vδ1 T cells is less well understood than that of the Vδ2 cells. However, recent findings shed new light on the ligand recognition by the Vδ1 TCR. It was demonstrated that the glycosphingolipid antigen sulfatide presented on the major histocompatibility complex (MHC) class I-like CD1d and CD1c molecules was recognized by Vδ1 cells (14–16). The myelin sheath, the target of the autoimmune attack in MS/EAE, is a rich source of sulfatide. This suggests that CD1c/d-restricted T cells could be activated by myelin-derived sulfatide in MS and, thereby, could be implicated in the onset of MS disease. Indeed, sulfatide reactive T cells have been found to be more frequent in the peripheral blood of MS patients than in healthy individuals (17), and studies in the mouse model demonstrated that sulfatide reactive CD1d-restricted T cells accumulated in the CNS during EAE (18). Taken together, these data indicate that Vδ1 cells, which include sulfatide reactive cells, may be involved in MS disease.

Here, we have investigated Vδ1 cells for their activation state and effector cell characteristics and compared them to other T cell subsets in newly diagnosed treatment naïve MS patients and patients treated with the disease-modifying therapy natalizumab (NTZ).

## Materials and Methods

### Experimental Design

Newly diagnosed MS patients (new-MS) diagnosed using the revised McDonald criteria (19) (*n* = 36) were enrolled at the MS center of Sahlgrenska University Hospital, Gothenburg, Sweden. Patients with any other chronic ailment, active disease, or receiving any disease-modifying treatment at the time of sampling were excluded. Treated MS patients (*n* = 25) receiving 300 mg NTZ intravenously once monthly were also included. Some (*n* = 7) new-MS patients were investigated before treatment and after 1–6 monthly doses of NTZ. Age- and gender-matched healthy blood donors (HD; *n* = 59) who visited the Department of Transfusion Medicine, Sahlgrenska University Hospital, served as controls. Demographics and clinical features of subjects are shown in Table 1.

**Table 1:** **Demographic and clinical features of newly diagnosed multiple sclerosis (new-MS) patients and healthy controls**.

	New-MS patients (*n* = 36)	Healthy donors (control) (*n* = 59)
**Age, years**		
Range	18–62	20–67
Mean ± SEM	35.4 ± 1.4	37.2 ± 1.6
**Gender, number (%)**		
Male	10 (27.7)	23 (39.0)
Female	26 (72.2)	36 (61.0)
**MRI T1 Gd^+^ lesions, number**		
Range	0–15	NA
Mean ± SEM	2.92 ± 0.61	NA
**MRI T2 lesions, number**		
Range	2–20	NA
Mean ± SEM	12.41 ± 1.10	NA
**EDSS**		
Range	0–6	NA
Mean ± SEM	2.14 ± 0.19	NA
**CSF IgG index**		
Range	0.44–3.09	NA
Mean ± SEM	1.20 ± 0.15	NA
**CSF OCB, number**		
Present	35	NA
Absent	1	NA

*CSF, cerebrospinal fluid; EDSS, expanded disability status scale; Gd^+^, gadolinium contrast enhancing; MRI, magnetic resonance imaging; NA, not applicable; OCB, oligoclonal bands*.

### Sample Preparation

Peripheral blood and CSF were always obtained for clinical purposes. The spinal tap and the CSF samples were handled according to the consensus protocol of the BioMS-EU network for CSF biomarker research in MS (20). Blood samples from patients (from patients on NTZ treatment just before the next dose) and healthy donors (HDs) were collected in heparinized tubes. Blood was diluted to 1:1 with sterile phosphate-buffered saline [(PBS); pH 7.2]. PBMCs were isolated by density gradient centrifugation using Ficoll-Paque (density: 1.077 g/mL; GE Healthcare). Cell viability was determined by Trypan Blue dye exclusion. The CSF lymphocyte cellularity was 9,850 ± 1,986/mL.

### Flow Cytometry

Immunophenotyping of cells was performed using 14-color flow cytometry. The following fluorochrome-conjugated anti-human monoclonal antibodies (mAbs) were used: anti-CD4 (clone OKT4), anti-CD8a (RPA-T8), anti-CD161 (HP-3G10), anti-TCRγδ (B1), anti-CCR4 (D8SEE) (all purchased from eBioscience), anti-CD3ϵ (OKT3), anti-CD19 (HIB19), anti-CD27 (O323), anti-CD28 (CD28.2), anti-CD45RA (HI100), anti-CD45RO (UCHL1), anti-CD49d (9F10), anti-CD69 (FN50), anti-TCRγδ (B1), anti-TCR Vα7.2 (3C10), anti-CCR6 (G034E3), anti-CCR7 (G043H7), anti-CXCR3 (G025H7) (all purchased from BioLegend), and anti-TCR Vδ1 (TS8.2) (Thermo Fisher Scientific). PBS-57 (an analog of α-galactosylceramide)-loaded and unloaded human CD1d tetramers were kindly provided by National Institutes of Health Tetramer Core Facility. Cells were stained with CD1d tetramers for 45 min at room temperature and then with surface mAbs for 30 min at 4°C. Cells were always stained with LIVE/DEAD^®^ aqua stain (Life Technologies) and Fc receptor binding inhibitor (eBioscience). Staining with the anti-TCRγδ (B1) mAb results in the appearance of two TCRγδ cell populations when plotted against CD3 or Vδ1 as noted before (21).

To stain intracellular cytokines and granzyme-B (GrB), PBMCs were stimulated with PMA (50 ng/mL) and ionomycin (500 ng/mL) (Sigma-Aldrich) in the presence of Brefeldin A (eBioscience) for 4 h at 37°C. Cells were surface stained as described above, fixed, and permeabilized using intracellular fixation and permeabilization buffer (eBioscience), and stained with anti-IFN-γ (4S.B3), anti-IL-10 (JES3-9D7), anti-IL-17A (BL168), anti-GrB (GB11) mAbs (BioLegend), and anti-TNF-α (MAb11) (eBioscience). Fluorescence minus one was used as background control (Figure S1A in Supplementary Material). Transcription factors were stained *ex vivo* with anti-T-bet (4B10) (BioLegend) and anti-RORγt (AFKJS-9) mAbs using Foxp3 staining buffer (eBioscience). Data were acquired using an LSRII (BD Biosciences) cytometer, and analyzed using FlowJo v.10 software (Tree Star).

### Neurofilament Light Chain (NFL) and Glial Fibrillary Acidic Protein (GFAP) Analyses

Neurofilament light chain concentration in CSF was measured with a sensitive sandwich enzyme-linked immunosorbent assay (ELISA) (NF-light^®^ ELISA kit, UmanDiagnostics AB, Umeå, Sweden). Intra- and inter-assay coefficients of variation were below 10%. The lower limit of quantification of the assay was 31 pg/mL. The GFAP concentration was measured by ELISA, as previously described (22). The absorbance was read at 490 nm, and the sensitivity of the GFAP assay was 16 pg/mL. Both assays were conducted at the Clinical Neurochemistry Laboratory (Sahlgrenska University Hospital) according to protocols approved by the Swedish Board for Accreditation and Conformity Assessment.

### Magnetic Resonance Imaging (MRI)

Diagnostic MRI scans of brain and cervical column were performed with 3.0-T machine using 1–3 mm thickness according to the Swedish guidelines (23). A standard protocol for MS was used with T1 3D-weighted sequence following a dose of intravenous gadolinium contrast, T2-weighted sequence, and 3D fluid-attenuated inversion recovery sequence. In all patients included in the study, the MRI demonstrated dissemination of lesions in space and time, thus fulfilling the revised McDonald criteria for MS diagnosis (19).

### Statistical Analysis

A Gaussian distribution was first assessed using D’Agostino and Pearson omnibus normality test, and parametric or non-parametric tests were carried out using unpaired *t*-test with Welch’s correction or Mann–Whitney *U*-test (two-tailed) for unpaired data and paired *t*-test or Wilcoxon matched-pairs signed rank test (two-tailed) for paired data to assess the differences between two groups, as specified in the figure legends. Correlation analyses were performed using Spearman’s non-parametric correlation. Results are presented as mean ± SEM, and *P*-values <0.05 were considered to be statistically significant. All statistical tests were performed using Prism GraphPad Software, version 6 (La Jolla, CA, USA).

### Study Approval

The Regional Ethical Review Board in Gothenburg, Sweden, approved this study, and written informed consent was obtained from each study subject.

## Results

### The TCR Vδ1 Subset in CSF of New-MS Patients

**Figure 1:**
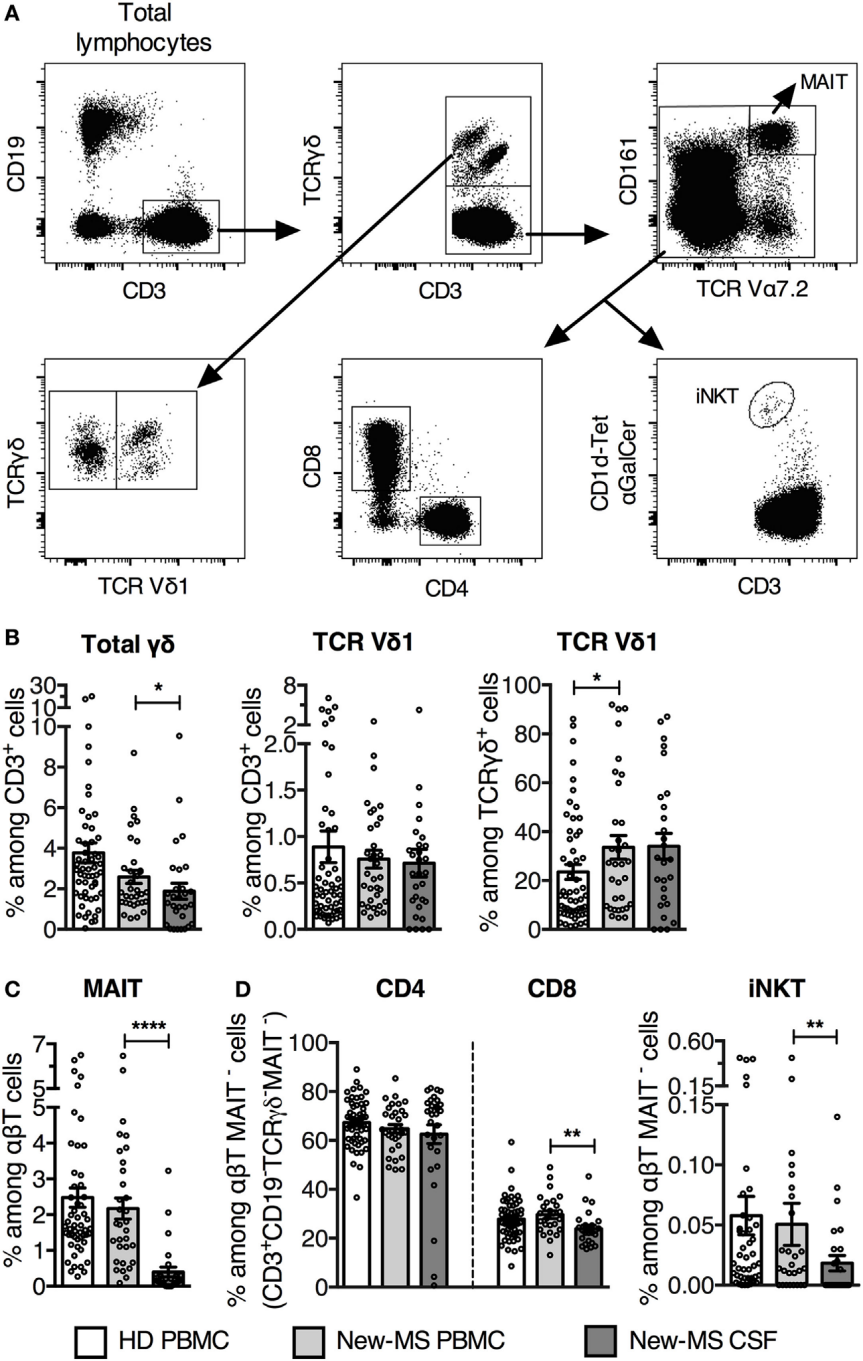
**Increased TCR Vδ1 T cell subset among γδ cells in newly diagnosed multiple sclerosis (new-MS) patients**. **(A)** Representative flow cytometric dot plots of healthy donors (HDs) PBMC showing gating strategy to define different T cell populations. TCR γδ^+^ T cells were first gated among CD19^−^CD3^+^ T cells (upper left and middle plots) and subdivided into Vδ1^+^ (Vδ1) and Vδ1^−^ (Vδ1^−^) T cells (lower left plot). After that, mucosal-associated invariant T (MAIT) cells were gated out from the TCR γδ^−^ CD3^+^ population (upper right plot), and thereafter CD4, CD8, and αGalCer-loaded CD1d-tetramer (CD1d-tet αGalCer) positive cells [invariant natural killer T (iNKT) cells] were gated among the remaining cells (lower row, middle and right plots). **(B)** Total TCRγδ cells were determined among CD3^+^CD19^−^ cells (referred to as CD3^+^ cells) (left), and TCR Vδ1 cells were represented among CD3^+^ cells (middle) and among TCRγδ^+^ cells (right). **(C)** Frequency of MAIT cells among αβT (CD3^+^CD19^−^ TCRγδ^−^) cells. **(D)** Frequencies of CD4, CD8, and iNKT cells within the CD3^+^CD19^−^TCRγδ^−^MAIT^−^ population (referred as αβT MAIT^−^ cells). Bars represent mean ± SEM (HD, *n* = 55; new-MS, *n* = 34). *P < 0.05; **P < 0.01; ****P < 0.0001. Unpaired *t*-test with Welch’s correction or Mann–Whitney *U*-test (two-tailed) between HD and new-MS and paired *t*-test or Wilcoxon matched-pairs signed rank test (two-tailed) between blood and cerebrospinal fluid (CSF) samples from new-MS were used to assess significance.

We first determined the frequencies of Vδ1 cells and different innate-like and conventional T lymphocyte populations (Figure 1A) in freshly provided CSF and PBMC from new-MS and PBMC from HD. It is important to note that none of the new-MS patients had received any treatment for MS disease, excluding prior immunomodulatory effects due to such treatment. We observed a wide range of γδ cell frequencies in PBMC of both new-MS and HD, without significant differences between the groups (Figure 1B). In new-MS patients, CSF had slightly lower frequencies than PBMC of total γδ cells among CD3^+^ cells. The size of the Vδ1 T subset among CD3^+^ cells in PBMC from new-MS patients did not show a significant difference compared with HD, while the frequency of Vδ1 cells among γδ cells was slightly increased in new-MS PBMC compared to HD PBMC. In new-MS individuals, the proportion of the Vδ1 cell subset was the same in CSF as in PBMC, suggesting that Vδ1 cells may enter the CNS in MS, in accordance with previous studies (13). Thus, we found that Vδ1 cells were present in CSF from new-MS patients, at the same levels as those found in MS PBMC.

We analyzed two additional subsets of innate-like T lymphocytes, mucosal-associated invariant T (MAIT) cells, and invariant natural killer T (iNKT) cells, which have been implicated in MS and EAE (24, 25). MAIT cells are activated by microbial and endogenous vitamin B metabolites presented on the MHC class I-like molecule MR1 and are defined by high expression of CD161 and an invariant TCR Vα7.2. The frequency of MAIT cells was similar in PBMC from new-MS and HD, but was several fold lower in new-MS CSF (Figure 1C), in agreement with a recent study (26). iNKT cells are α-galactosylceramide (αGalCer) reactive, CD1d-restricted TCRαβ cells that use an invariant Vα24 TCR α-chain. Employing αGalCer-loaded CD1d-tetramers, we observed that frequencies of iNKT cells in PBMC from new-MS patients and HD were similar, while they were significantly lower in new-MS CSF (Figure 1D). Finally, among conventional T cells, the proportion of CD4 T cells was comparable in HD and new-MS PBMC and CSF, while CD8 T cells were slightly lower in new-MS CSF than in PBMC (Figure 1D).

Thus, in MS patients, Vδ1 T cells were present in CSF at frequencies equal to PBMC, while the two other innate-like T cell populations, MAIT, and iNKT cells, had severely reduced representation in CSF.

### Vδ1 Cells from New-MS Patient CSF Had an Altered Phenotype

**Figure 2:**
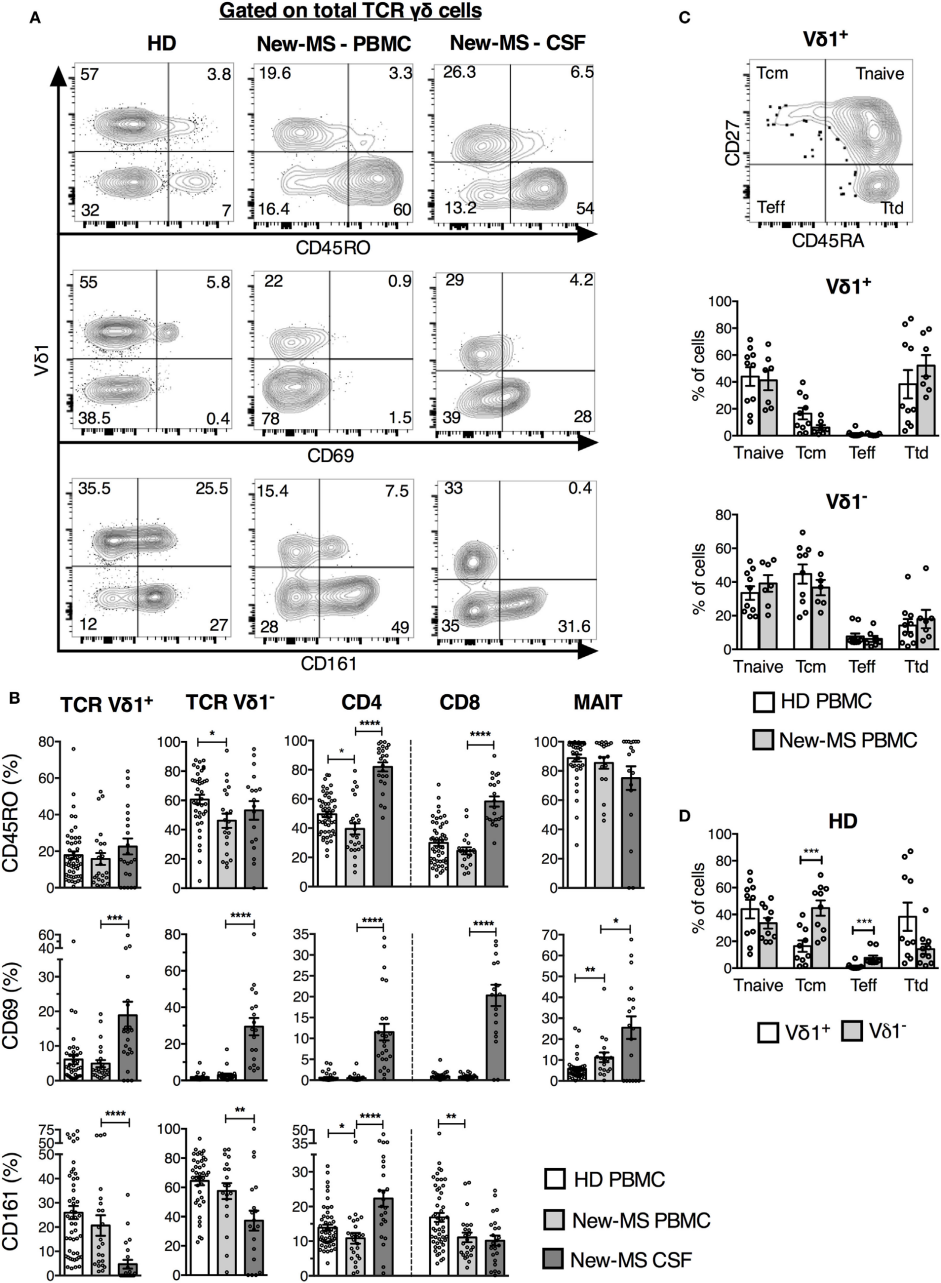
**Vδ1 cells from newly diagnosed multiple sclerosis (new-MS) patients had a phenotype of recent activation**. **(A)** T cells were gated as in Figure 1, and expression of indicated markers was determined. Representative flow cytometric plots are shown for total γδ cells. **(B)** Cells within each T cell subset expressing CD45RO [upper panel, healthy donors (HDs) *n* = 50, new-MS patients *n* = 24], CD69 (middle panel, HD *n* = 40, new-MS *n* = 24), and CD161 (lower panel, HD *n* = 50, new-MS *n* = 24) were determined. **(C)** Based on the expression of CD45RA and CD27 (representative dot plot for Vδ1 cells from HD), Vδ1 cells were divided into naïve (Tnaive, CD45RA^+^CD27^+^), central memory (Tcm, CD45RA^−^CD27^+^), effector (Teff, CD45RA^−^CD27^−^), and terminally differentiated effector (Ttd, CD45RA^+^CD27^−^) cells (30). Graphs compare values for Vδ1 and Vδ1^−^ T cells in PBMC from HD (*n* = 10) and new-MS patients (*n* = 7). **(D)** Vδ1 and Vδ1^−^ T cells from HD (*n* = 10). Bars represent mean ± SEM. *P < 0.05; **P < 0.01; ***P < 0.001; ****P < 0.0001. Unpaired *t*-test with Welch’s correction or Mann–Whitney *U*-test (two-tailed) for HD versus new-MS PBMC and paired *t*-test or Wilcoxon matched-pairs signed rank test (two-tailed) for new-MS PBMC versus cerebrospinal fluid (CSF) were used to assess significance.

We next investigated the expression of surface markers that could identify T cells engaged in immune responses in MS patients (Figure 2). Cells were stained for CD69, first defined as an early activation marker but also expressed on leukocytes at the site of inflammation, the late activation/memory marker CD45RO, and CD161, a receptor with co-regulatory function and associated with IL-17 production (27). CD161 is found on NK cells and subpopulations of T cells, such as MAIT cells and the majority of γδ T cells expressing the Vδ2 TCR. Throughout, we have compared Vδ1^+^ T cells (Vδ1 T) with the rest of γδ T cells (here termed Vδ1^−^ T cells), and with the other T cell subsets. Peripheral blood γδ T cells predominantly use the Vδ2 TCR-chain, thus the majority of γδ T cells here defined as Vδ1^−^ are likely to express the Vδ2 segment (28, 29). iNKT cells were omitted from these analyses due to limiting cell numbers in several samples.

All T cell populations, including γδ T cell subsets, demonstrated an increased proportion of CD69^+^ cells in CSF (an average of 15–30% in the different cell populations) compared to PBMC (average of 7% or less) from new-MS patients, indicating a memory/tissue resident T cell phenotype (Figures 2A,B). Moreover, CD161 levels were reduced on both γδ cell subsets in CSF, in particular on Vδ1 T cells, while CD161 instead was found on a higher proportion of CSF CD4^+^ T cells (Figures 2A,B). This further illustrates that these cells might be functionally altered.

Besides a minor reduction in CD45RO expression among Vδ1^−^ T cells in new-MS PBMC, no alterations in the expression of the analyzed surface markers by γδ T cell subsets were found between new-MS and HD PBMC. CD45RO was also slightly lower in frequency in new-MS CD4 T cells than in HD CD4 T cells, and CD8 T cells from patients had fewer cells expressing CD161 than CD8 cells from HD (Figure 2B).

We finally tested an additional pair of surface markers, CD27, and CD45RA, hoping to distinguish activated γδ cells in PBMC of MS patients. It has been proposed that γδ cells can be divided into different naïve/memory/effector subsets by their differential expression of these markers (30). Accordingly, γδ cells were divided into subsets as shown in Figure 2C. The expression of these markers by Vδ1 and Vδ1^−^ T cells in PBMC did not differ markedly between HD and new-MS patients. In both groups, Vδ1 cells were comprised primarily of cells with phenotypes of naïve and terminally differentiated cells, while Vδ1^−^ cells had mostly naïve and central memory phenotypes (Figures 2C,D).

Taken together, the analysis of a set of activation/memory markers demonstrated that all T cell subsets in CSF contained elevated frequencies of recently activated cells with potentially altered function compared to PBMC, consistent with an active involvement in CNS autoimmunity. By contrast, neither of these markers revealed an increase in activated T cells in the peripheral blood of new-MS patients, with the exception of minor changes found in CD4 and CD8 T cells.

### IFN-γ Production Was Significantly Increased Specifically in Vδ1 Cells in New-MS Patients

**Figure 3:**
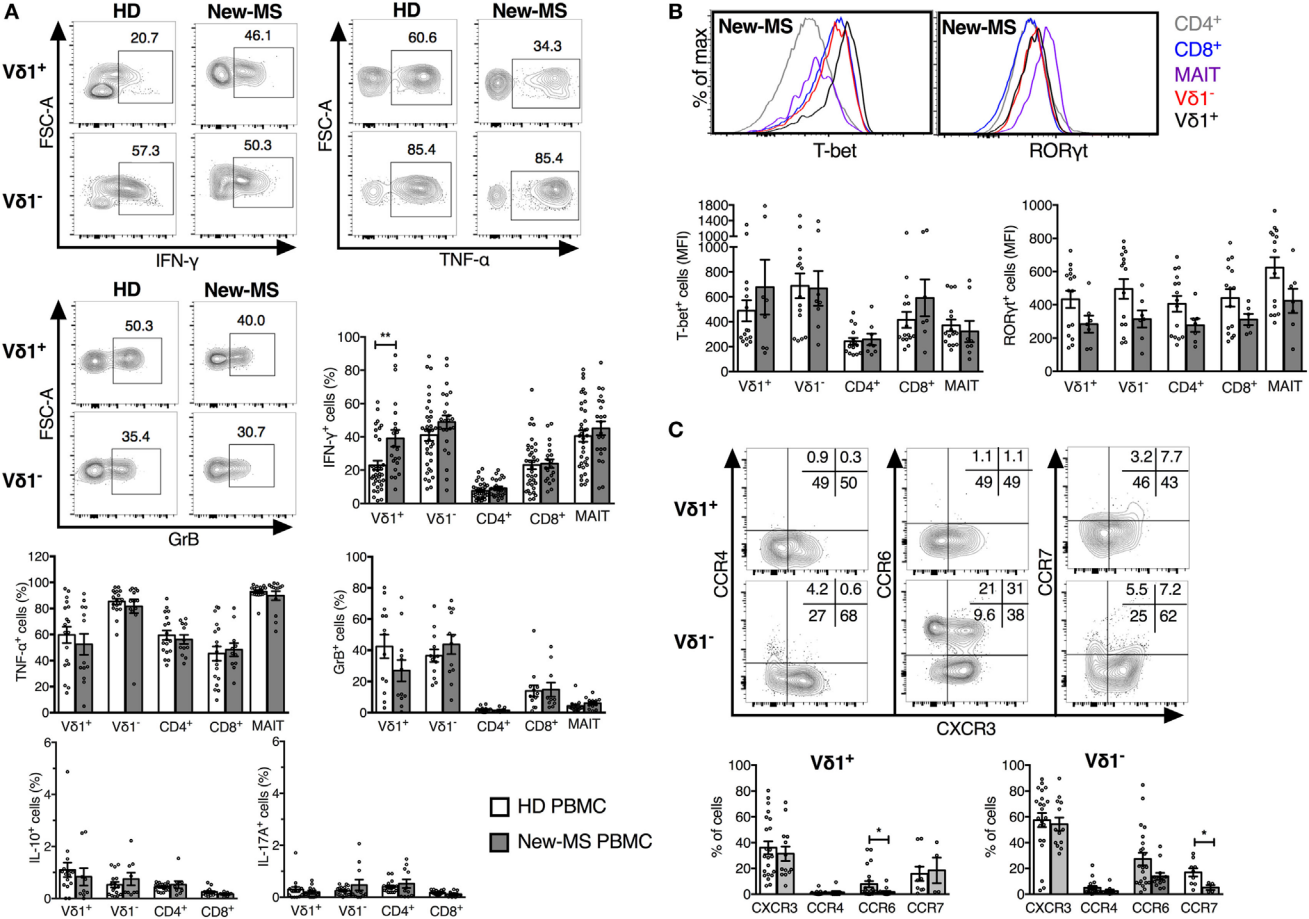
**Increased IFN-γ production by Vδ1 cells in PBMC from newly diagnosed multiple sclerosis (new-MS) patients**. T cell subsets were defined as in Figure 1 and analyzed by flow cytometry for the expression of cytokines **(A)**, transcription factors **(B)**, and chemokine receptors **(C)**. Cells were stimulated as described in Section “Materials and Methods” before staining **(A)** or were stained for directly *ex vivo* **(B,C)**. Representative plots are shown for Vδ1 and Vδ1^−^ T cells from healthy donors (HDs) and new-MS as indicated **(A)**, Vδ1, Vδ1^−^, mucosal-associated invariant (MAIT), and conventional CD4/8 T cells from new-MS patients **(B)**, and for Vδ1 and Vδ1^−^ T cells from HD **(C)**. Bars represent mean ± SEM of cells expressing IFN-γ (HD *n* = 35, new-MS *n* = 21), TNF-α (HD *n* = 18, new-MS *n* = 13), granzyme-B (GrB) (HD *n* = 13, new-MS *n* = 12), IL-10 and IL-17A (HD *n* = 16, new-MS *n* = 9), CXCR3 and CCR6 (HD *n* = 22, new-MS *n* = 13), CCR4 (HD *n* = 17, new-MS *n* = 9), CCR7 (HD *n* = 9, new-MS *n* = 4), and median fluorescent intensity (MFI) for RORγt and T-bet (HD *n* = 15, new-MS *n* = 8). *P < 0.05, **P < 0.01. Unpaired *t*-test with Welch’s correction or Mann–Whitney *U*-test (two-tailed) was used to assess significance.

Having failed to find marked differences in activation markers on peripheral blood T cells from MS patients compared to HD, we turned to a wide analysis of putative function (Figure 3). Strikingly, we demonstrate a significant increase in IFN-γ production in Vδ1 cells from new-MS patients compared to those from HD (39.1 versus 22.9%, *P* = 0.0079) (Figure 3A). A high proportion of Vδ1 T cells also produced TNF-α and GrB (Figure 3A), and the Vδ1 population displayed high levels of T-bet, a transcription factor associated with IFN-γ production (Figure 3B). In fact, the vast majority of Vδ1 cells producing IFN-γ also produced TNF-α, while there were both single and double producers of GrB and IFN-γ (data not shown). The increased IFN-γ production in new-MS patients was specific for Vδ1 cells, as other T cell populations did not exhibit differences in the production of IFN-γ (Figure 3A). The production of both IL-10 and IL-17A was very low in all T cell subsets from both HD and new-MS patients (Figure 3A). CCR6, known to be expressed on Th17 cells, was reduced to very low levels on Vδ1 cells in new-MS PBMC, consistent with negligible levels of IL-17, low RORγt, and an elevated IFN-γ expression. By contrast, the percentage of Vδ1 cells that expressed CXCR3, CCR4, and CCR7 remained unaltered (Figure 3C). Our results clearly demonstrate that, in peripheral blood of new-MS patients, Vδ1 cells selectively had a significantly elevated capacity to produce IFN-γ.

Previous studies comparing the cytokine profile of γδ T cell subsets in HD suggest that Vδ2 cells are more inflammatory (produce more IFN-γ and IL-17) while Vδ1 cells are more regulatory (produce more IL-10) (31, 32). This was verified by our data and is illustrated in Figures S1B,C in Supplementary Material, showing results from HD and new MS-patients from Figure 3 to allow a direct comparison between Vδ1 and Vδ1^−^ T cells. By contrast, in new-MS patients, the frequencies of Vδ1 cells producing IFN-γ and IL-10 were not significant different from those of Vδ1^−^ T cells.

Vδ1^−^ cells demonstrated higher frequencies of cells producing IFN-γ and TNF-α compared to Vδ1 cells, while the levels of GrB were similar. T-bet expression was not significantly different although Vδ1^−^ T cells showed a tendency of higher levels. By contrast, Vδ1 cells contained significantly higher frequencies of IL-10-producing cells, although still at low levels, than the Vδ1^−^ T cell subset. Expression of RORγt and IL-17A was very low in both γδ cell subsets from HD, but Vδ1^−^ cells had an increased fraction of cells expressing CCR6, which is associated with IL-17 production, as well as a slight increase in CCR4.

Thus, our broad analysis of functional attributes of T cells in new-MS peripheral blood could identify that Vδ1 T cells uniquely had a significantly increased capacity to produce IFN-γ, implicating Vδ1 cells in the etiology of MS disease.

### IFN-γ^+^ Vδ1 Cells in Peripheral Blood Correlated with Clinical Scores for Disease Activity, Inflammation, and Axonal Damage in MS Patients

**Figure 4:**
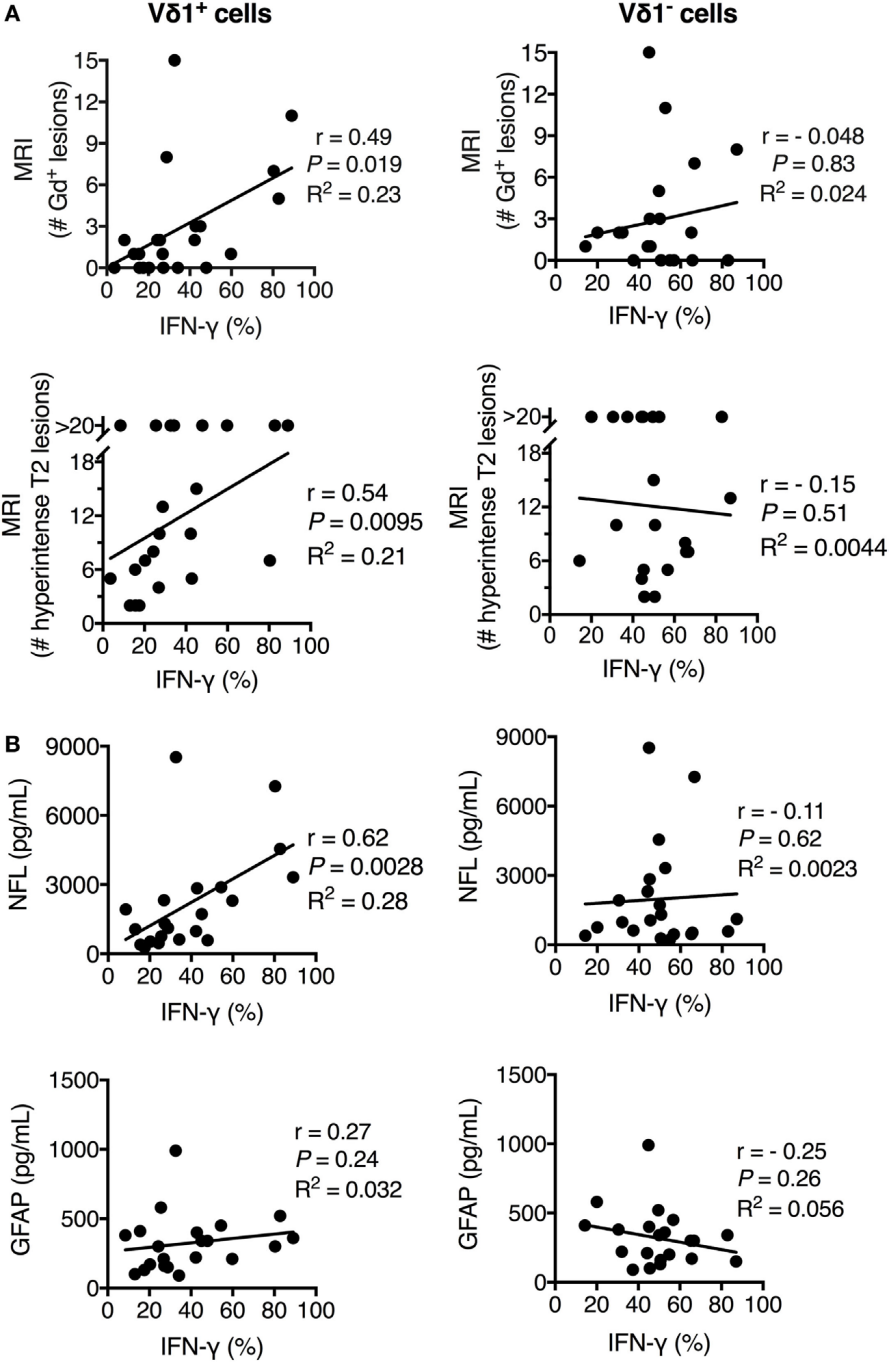
**Frequency of IFN-γ^+^ Vδ1 cells positively correlated with multiple sclerosis (MS) disease activity, inflammation, and central nervous system (CNS) damage in newly diagnosed multiple sclerosis patients**. The expression of IFN-γ (as determined in Figure 3) in Vδ1 (left plots) and Vδ1^−^ (right plots) T cells was correlated with T1 gadolinium contrast-enhanced (Gd^+^) MRI lesions (upper plots) and T2 lesions (lower plots) **(A)** and cerebrospinal fluid concentrations of biomarkers for CNS damage (NFL, middle plots, and GFAP, lower plots) **(B)** (*n* = 21). *r* is Spearman’s correlation coefficient. NFL, neurofilament light chain; GFAP, glial fibrillary acidic protein; MRI, magnetic resonance imaging. Spearman non-parametric correlation test was used. *R*^2^ indicates the Goodness of Fit line (calculated using linear regression correlation).

To further substantiate the link between Vδ1 cells and MS disease, we extended the analysis to biomarkers of disease activity and neuronal damage. We had noted that, although the average frequency of IFN-γ-producing Vδ1 cells in PBMC of new-MS patients was significantly increased compared to that of HD, there was a high variability in the values between individual new-MS patients. We hypothesized that this diversity could be related to heterogeneity in disease activity in the patient group. We, therefore, compared our results with clinical data from gadolinium contrast-enhanced (Gd^+^) MRI investigations that detect active MS, i.e., T1 gadolinium contrast-enhanced (Gd^+^) lesions. The frequency of IFN-γ^+^ Vδ1 cells from PBMC correlated significantly with the number of T1 Gd^+^ lesions (*P* = 0.019, Figure 4A, upper graph). We also found a correlation with the number of T2 lesions (*P* = 0.0095, Figure 4A, lower graph). Moreover, there was a strong correlation between the percentage of IFN-γ^+^ Vδ1 cells from PBMC with CSF concentrations of neurofilament light protein NFL (*P* = 0.0028) indicating axonal damage. By contrast, there was no significant correlation with CSF GFAP (*P* = 0.24), a marker of astrocyte damage and astrogliosis (Figure 4B). No correlation with these parameters was observed for Vδ1 cells producing other cytokines nor for the production of IFN-γ (or other cytokines) in Vδ1^−^ T cells or the other T cell subsets (Figure 4 and data not shown). Thus, the level of IFN-γ production in Vδ1 T cells correlated positively with biomarkers of axonal damage and disease activity, and taken together, these data suggest that Vδ1 cells may have a disease-promoting function in early MS.

### Disease-Modifying Treatment Normalized Vδ1 Cell IFN-γ Production

**Figure 5:**
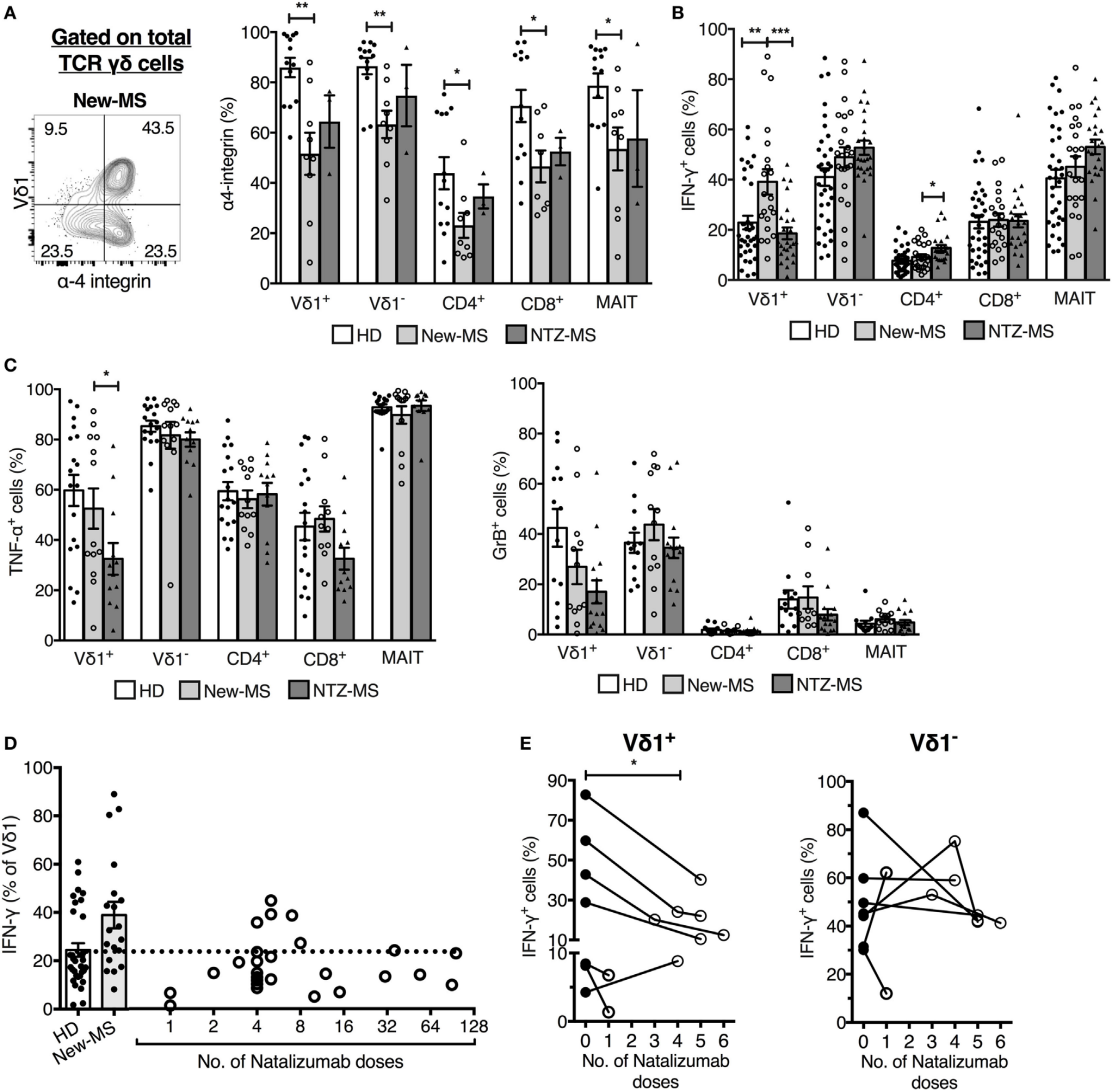
**Natalizumab (NTZ) treatment of multiple sclerosis (MS) patients normalized IFN-γ production in Vδ1 T cells**. **(A)** Expression of α-4 integrin on T cell populations (right) [healthy donors (HDs) *n* = 13, new-MS *n* = 9, and NTZ-MS *n* = 3]. Representative staining on total γδ cells from a new-MS patient (left). **(B,C)** Expression of IFN-γ, TNF-α, and granzyme-B (GrB) in PBMC from HD (IFN-γ *n* = 35, TNF-α *n* = 18, and GrB *n* = 13), new-MS (IFN-γ *n* = 21, TNF-α, and GrB *n* = 12), and NTZ-MS patients (IFN-γ *n* = 25, TNF-α *n* = 12, and GrB *n* = 15). **(D)** Frequency of IFN-γ producing Vδ1 cells in HD and new-MS patients (bars), and after different numbers of monthly NTZ treatments (1–96 treatments, circles representing individual patients; *n* = 25). **(E)** Frequencies of IFN-γ-producing Vδ1 (left) and Vδ1^−^ T cells (right) were analyzed before and after NTZ treatments (1–6 doses) of new-MS patients. Statistical difference was assessed between the sample taken before treatment and the first sample collected after treatment. Bars represent mean ± SEM. *P < 0.05, **P < 0.01, ***P < 0.001. Unpaired *t*-test with Welch’s correction or Mann–Whitney *U*-test (two-tailed) and paired *t*-test or Wilcoxon matched-pairs signed rank test (two-tailed) were used to assess significance. Note that data shown for IFN-γ production in **(D,E)** (first data point after treatment only) are included in **(B)**, and these data shown in **(E)** are included in **(D,B)** [for each patient in **(E)**, only the data points before treatment and after the first treatment are included].

We had access to MS patients who were undergoing treatment for the disease and could, therefore, explore in a second context the association of Vδ1 T cells and disease activity. MS patients treated with the disease-modifying agent NTZ, an antibody that blocks α4-integrin required for T lymphocyte entry across the blood–brain barrier (1), have a drastically reduced disease activity (33). We analyzed whether NTZ treatment had an effect on the IFN-γ production by Vδ1 cells by comparing patients treated with NTZ (NTZ-MS) with our cohorts (see Figure 3A) of new-MS patients and HD (Figure 5). We first established that Vδ1 cells, like other T cell subsets, have a high expression of α4-integrin (Figure 5A). In new-MS patients, the fraction expressing α4-integrin among T cells was variable, and on average, around 50% among Vδ1 cells. Strikingly, MS patients who had been treated with NTZ (NTZ-MS) had completely reduced frequencies of IFN-γ-producing Vδ1 cells to a level similar to HD. The treatment did not reduce IFN-γ production in any other T cell population analyzed, rather, there was an increase in IFN-γ production in CD4^+^ T cells after treatment (Figure 5B). NTZ treatment also reduced the fraction of Vδ1 cells producing TNF-α, and there was a trend, not significant in our data set, of reduction of GrB in the Vδ1 cell population (Figure 5C). The group of patients in Figure 5B that demonstrated normalized IFN-γ production in Vδ1 cells had been treated for various periods of time, from 1 to 96 monthly doses (illustrated in Figure 5D). Indeed, in individual patients among those who had been tested both before and after treatment, a significant reduction of IFN-γ production specifically in Vδ1 cells by NTZ treatment could be observed (*P* = 0.037, Figure 5E). By contrast, there was no consistent alteration of IFN-γ production in any other T cell subset after treatment. Thus, NTZ treatment of MS patients had an effect on IFN-γ production that was exclusive to Vδ1 cells, reinforcing the notion that Vδ1 T cells may be pathogenic and may promote early MS disease.

## Discussion

The early immune events that lead to the precipitation of RRMS are not well understood. To shed more light on the early processes of MS disease pathogenesis, here, we have investigated a T lymphocyte population, Vδ1 TCRγδ T cells, which belong to the innate-like lymphocytes characterized by rapid activation and early involvement during immune responses. The results demonstrate that activation of Th1-like Vδ1 cells correlates with disease activity and neuronal destruction early in MS pathogenesis and imply that Vδ1 cells may contribute to disease progression by the production of the proinflammatory cytokine IFN-γ.

Vδ1 cells were present in the CSF of new-MS patients and had an elevated expression of CD69 compared to PBMC. This was not unique for Vδ1 cells as CD69 was expressed on all T cell populations in CSF. CD69 has previously been found together with a memory phenotype also on T cells in CSF from individuals that have no inflammatory CNS disorders, suggesting that memory T cells can enter the CSF in healthy individuals and are involved in immune surveillance of the CNS (34). We found no alterations in the expression of surface markers by Vδ1 cells in PBMC from new-MS patients compared to HD; however, more detailed analysis demonstrated that new-MS patients harbored significantly higher frequencies than HD of circulating Vδ1 cells that exhibit IFN-γ production, without alterations in the expression of other cytokines. Other T cell subsets analyzed did not demonstrate any changes in cytokine production at the population level. Initially, IFN-γ was considered the key pathogenic cytokine in MS, but its role in the disease has been questioned during recent years (1, 35, 36). However, a number of findings support a disease-promoting role for this cytokine. Increased levels of IFN-γ are reported in CSF in MS, as well as in the spinal cords of mice with EAE (37), and recombinant IFN-γ treatment provoked relapses in RRMS patients in a clinical trial (38). In the EAE model, myelin-reactive Th1 cells adoptively transferred into mice induced the disease, and it was shown that Th1 cells drive a spontaneous MS disease model (39, 40). Further, a rapid transformation of Th17 cells into IFN-γ producers in the CNS of EAE mice was demonstrated using IL-17 fate-reporter mice (41), revealing plasticity of Th17 cells. A reconciling model has been put forward that emphasize the role of IFN-γ-producing T cells in the early phase of CNS autoimmunity, proposing that Th1 cells are required to infiltrate the non-inflamed CNS, facilitating the entry of Th17 cells (3). The IFN-γ-producing Vδ1 cells that we demonstrate in new-MS patients also produced TNF-α and to some extent GrB, but very low levels of IL-10 and IL-17. Consistent with this, Th1-like cytokine expression pattern, new-MS Vδ1 cells (like HD Vδ1 cells) were positive for Th1-associated T-bet and CXCR3, but were negative for RORγt expressed by Th17 cells. A previous study demonstrated that total γδ T cells in CSF have a larger fraction of CD161^high^ CCR6^+^ cells than found in PBMC (42). The fact that we find reduced display of CD161 on γδ T cells in new-MS CSF compared to PBMC is compatible with the data from Schirmer et al., as we analyzed cells positive for only this marker. Taken together, published data provide substantial evidence for a pathogenic role for IFN-γ in EAE/MS. The fact that we find an elevation of IFN-γ-producing Vδ1 cells in new-MS patient PBMC, therefore, implicate that these cells may contribute to disease pathogenesis. Future studies will be important to further characterize Vδ1 cells in CSF to strengthen this notion.

In further support of a causal role of Vδ1 cells in MS disease etiology, we found that the frequency of IFN-γ producing Vδ1 cells demonstrated a high correlation with clinical data and biomarkers of disease activity and axonal damage such as numbers of MRI lesions and levels of NFL in CSF. The association was particularly strong with NFL, a marker of axonal damage. The concentration of NFL is shown to be increased in CSF at all stages of MS, it is elevated with the presence of active MRI lesions, and the highest concentrations are found during acute relapses (43). Importantly, long-term follow-up studies have demonstrated that the NFL concentrations found at initial diagnosis of MS were predictive of disease severity and advancement to secondary progressive disease (44). Thus, our findings suggest that the Th1-skewed activation of Vδ1 cells is related to axonal damage.

Moreover, we found that treatment with the disease-modifying therapy NTZ completely reduced Vδ1 T cell IFN-γ production to normal levels. If the effect of NTZ on Vδ1 T cells was solely to prevent activated Vδ1 T cells from entering the CNS, it could be anticipated that cytokine production in this subset in PBMC would be increased after treatment, due to the accumulation of activated cells in the circulation, as found for conventional CD4^+^ T cells (45) and Figure 5. Instead, NTZ normalized the IFN-γ-producing capacity of Vδ1 cells. The reason for the differential effects on CD4^+^ T cells versus Vδ1 T cells is unknown and can only be speculated about. One possibility is that NTZ treatment results in a lower ability of Vδ1 cells to access their site of activation, Alternatively, Vδ1 T cells may have an inferior cell intrinsic capacity to maintain IFN-γ production long term (months) after stimulation. Either mechanism, or a combination, could result in the decreased IFN-γ production by Vδ1 T cells in treated patients. It is also possible that unknown indirect mechanisms cause the reduction in IFN-γ production by Vδ1 T cells after treatment. Integrin α4β1, targeted by NTZ in MS, is required for T lymphocyte transmigration across the blood–brain barrier, and in fact, regulates lymphocyte migration in all organs. In addition, α4-integrin is found on the cell surface associated with either integrin β1 or β7. Integrin α4β7 is essential for homing of T lymphocytes to the gut, and NTZ has, therefore, also been used for treatment of Crohn’s disease. It remains to be resolved whether the putative site of activation of Vδ1 T cells is in the CNS, or potentially another site, and whether this relates to early autoimmune activation in MS pathogenesis.

The most predominant location of Vδ1 cells is in the intestinal epithelium where they comprise around 70% of γδ lymphocytes (46). Intraepithelial T lymphocytes maintain tolerance and integrity of the epithelial barrier under homeostatic conditions and promote immune protection from pathogens. Vδ1 cells that express IFN-γ and IL-17 are expanded in the periphery during some infections (47–49). Vδ1 cells are also implicated in the immune response against cancers (50), and intestinal Vδ1 cells were found to be the major source of tumor-promoting IL-17 in human colorectal cancer (51). This is in stark contrast to healthy individuals, in which Vδ1 cells are described as having more of regulatory characteristics and less inflammatory functions compared to Vδ2 γδ cells (31, 32). Interestingly, intestinal Vδ1 cells are expanded in celiac disease, and Vδ1 cells with gut-homing potential appeared in peripheral blood following gluten challenge of celiac patients on gluten-free diet (46, 52). This illustrates the diverse functions that can be expressed by γδ cells in different immune responses (8) and establishes that Vδ1 cells in new-MS patients have a functional phenotype, expressing IFN-γ, but not IL-17, clearly distinct from these situations. It also points to a possible connection between activated systemic Vδ1 cells and an immune trigger at a mucosal site. Evidence is accumulating that alterations in the intestinal microflora can mediate an increased risk for autoimmune disease, including EAE and MS (1, 53–55). It is evident from animal experiments that effects of the gut flora on T lymphocytes residing in intestinal mucosal tissues influence autoimmune responses in remote tissues. In this perspective, Vδ1 cells residing in the intestinal epithelium, thus located at the first tissue barrier in contact with gut bacteria and being sentinels of microbial alterations, should be considered in the interplay between microbiota and the immune system leading to autoimmune disease such as MS. Our finding that new-MS patients have a significant increase in IFN-γ-producing circulating Vδ1 cells reinforces this notion.

## Author Contributions

SLC and JL designed the study; AKS conducted experiments; MA, LN, CM, and JL were responsible for clinical assessment and patient selection, analysis of biomarkers, and MRI; HZ was responsible for analysis of biomarkers. All the authors analyzed data, and SLC and AKS wrote the manuscript with major contributions from all other authors.

## Conflict of Interest Statement

The authors declare that the research was conducted in the absence of any commercial or financial relationships that could be construed as a potential conflict of interest.

## Abbreviations

CSF, cerebrospinal fluid; EAE, experimental autoimmune encephalomyelitis; GFAP, glial fibrillary acidic protein; HDs, healthy donors; iNKT, invariant natural killer T; MAIT, mucosal-associated invariant T; MS, multiple sclerosis; new-MS, newly diagnosed MS patients; NFL, neurofilament light chain; NTZ, natalizumab.

## References

[1] Steinman L. Immunology of relapse and remission in multiple sclerosis. Annu Rev Immunol (2014) 32:257–81. 10.1146/annurev-immunol-032713-120227 24438352

[2] Bettelli E Sullivan B Szabo SJ Sobel RA Glimcher LH Kuchroo VK. Loss of T-bet, but not STAT1, prevents the development of experimental autoimmune encephalomyelitis. J Exp Med (2004) 200(1):79–87. 10.1084/jem.20031819 15238607 PMC2213316

[3] O’Connor RA Prendergast CT Sabatos CA Lau CW Leech MD Wraith DC Cutting edge: Th1 cells facilitate the entry of Th17 cells to the central nervous system during experimental autoimmune encephalomyelitis. J Immunol (2008) 181(6):3750–4. 10.4049/jimmunol.181.6.3750 18768826 PMC2619513

[4] Ghoreschi K Laurence A Yang XP Tato CM McGeachy MJ Konkel JE Generation of pathogenic T(H)17 cells in the absence of TGF-beta signalling. Nature (2010) 467(7318):967–71. 10.1038/nature09447 20962846 PMC3108066

[5] Codarri L Gyulveszi G Tosevski V Hesske L Fontana A Magnenat L RORgammat drives production of the cytokine GM-CSF in helper T cells, which is essential for the effector phase of autoimmune neuroinflammation. Nat Immunol (2011) 12(6):560–7. 10.1038/ni.2027 21516112

[6] El-Behi M Ciric B Dai H Yan Y Cullimore M Safavi F The encephalitogenicity of T(H)17 cells is dependent on IL-1- and IL-23-induced production of the cytokine GM-CSF. Nat Immunol (2011) 12(6):568–75. 10.1038/ni.2031 21516111 PMC3116521

[7] Lee Y Awasthi A Yosef N Quintana FJ Xiao S Peters A Induction and molecular signature of pathogenic TH17 cells. Nat Immunol (2012) 13(10):991–9. 10.1038/ni.2416 22961052 PMC3459594

[8] Vantourout P Hayday A Six-of-the-best: unique contributions of gammadelta T cells to immunology. Nat Rev Immunol (2013) 13(2):88–100. 10.1038/nri3384 23348415 PMC3951794

[9] Spahn TW Issazadah S Salvin AJ Weiner HL. Decreased severity of myelin oligodendrocyte glycoprotein peptide 33–35-induced experimental autoimmune encephalomyelitis in mice with a disrupted TCR delta chain gene. Eur J Immunol (1999) 29(12):4060–71. 10.1002/(SICI)1521-4141(199912)29:12<4060::AID-IMMU4060>3.0.CO;2-S 10602017

[10] Blink SE Miller SD. The contribution of gammadelta T cells to the pathogenesis of EAE and MS. Curr Mol Med (2009) 9(1):15–22. 10.2174/156652409787314516 19199938 PMC2845639

[11] Sutton CE Lalor SJ Sweeney CM Brereton CF Lavelle EC Mills KH. Interleukin-1 and IL-23 induce innate IL-17 production from gammadelta T cells, amplifying Th17 responses and autoimmunity. Immunity (2009) 31(2):331–41. 10.1016/j.immuni.2009.08.001 19682929

[12] Wucherpfennig KW Newcombe J Li H Keddy C Cuzner ML Hafler DA. Gamma delta T-cell receptor repertoire in acute multiple sclerosis lesions. Proc Natl Acad Sci U S A (1992) 89(10):4588–92. 10.1073/pnas.89.10.4588 1374907 PMC49128

[13] Shimonkevitz R Colburn C Burnham JA Murray RS Kotzin BL. Clonal expansions of activated gamma/delta T cells in recent-onset multiple sclerosis. Proc Natl Acad Sci U S A (1993) 90(3):923–7. 10.1073/pnas.90.3.923 8430106 PMC45782

[14] Bai L Picard D Anderson B Chaudhary V Luoma A Jabri B The majority of CD1d-sulfatide-specific T cells in human blood use a semiinvariant Vdelta1 TCR. Eur J Immunol (2012) 42(9):2505–10. 10.1002/eji.201242531 22829134 PMC3743557

[15] Luoma AM Castro CD Mayassi T Bembinster LA Bai L Picard D Crystal structure of Vdelta1 T cell receptor in complex with CD1d-sulfatide shows MHC-like recognition of a self-lipid by human gammadelta T cells. Immunity (2013) 39(6):1032–42. 10.1016/j.immuni.2013.11.001 24239091 PMC3875342

[16] Roy S Ly D Castro CD Li NS Hawk AJ Altman JD Molecular analysis of lipid-reactive Vdelta1 gammadelta T cells identified by CD1c tetramers. J Immunol (2016) 196(4):1933–42. 10.4049/jimmunol.1502202 26755823 PMC4744554

[17] Shamshiev A Donda A Carena I Mori L Kappos L De Libero G. Self glycolipids as T-cell autoantigens. Eur J Immunol (1999) 29(5):1667–75. 10.1002/(SICI)1521-4141(199905)29:05<1667::AID-IMMU1667>3.0.CO;2-U 10359121

[18] Jahng A Maricic I Aguilera C Cardell S Halder RC Kumar V. Prevention of autoimmunity by targeting a distinct, noninvariant CD1d-reactive T cell population reactive to sulfatide. J Exp Med (2004) 199(7):947–57. 10.1084/jem.20031389 15051763 PMC2211873

[19] Polman CH Reingold SC Banwell B Clanet M Cohen JA Filippi M Diagnostic criteria for multiple sclerosis: 2010 revisions to the McDonald criteria. Ann Neurol (2011) 69(2):292–302. 10.1002/ana.22366 21387374 PMC3084507

[20] Teunissen CE Petzold A Bennett JL Berven FS Brundin L Comabella M A consensus protocol for the standardization of cerebrospinal fluid collection and biobanking. Neurology (2009) 73(22):1914–22. 10.1212/WNL.0b013e3181c47cc2 19949037 PMC2839806

[21] Yokobori N Schierloh P Geffner L Balboa L Romero M Musella R CD3 expression distinguishes two gammadeltaT cell receptor subsets with different phenotype and effector function in tuberculous pleurisy. Clin Exp Immunol (2009) 157(3):385–94. 10.1111/j.1365-2249.2009.03974.x 19664147 PMC2745033

[22] Rosengren LE Wikkelso C Hagberg L. A sensitive ELISA for glial fibrillary acidic protein: application in CSF of adults. J Neurosci Methods (1994) 51(2):197–204. 10.1016/0165-0270(94)90011-6 8051950

[23] Vagberg M Axelsson M Birgander R Burman J Cananau C Forslin Y Guidelines for the use of magnetic resonance imaging in diagnosing and monitoring the treatment of multiple sclerosis: recommendations of the Swedish Multiple Sclerosis Association and the Swedish Neuroradiological Society. Acta Neurol Scand (2017) 135(1):17–24. 10.1111/ane.12667 27558404 PMC5157754

[24] Treiner E Liblau RS. Mucosal-associated invariant T cells in multiple sclerosis: the jury is still out. Front Immunol (2015) 6:503. 10.3389/fimmu.2015.00503 26483793 PMC4588106

[25] Van Kaer L Wu L Parekh VV Natural killer T cells in multiple sclerosis and its animal model, experimental autoimmune encephalomyelitis. Immunology (2015) 146(1):1–10. 10.1111/imm.12485 26032048 PMC4552496

[26] Willing A Leach OA Ufer F Attfield KE Steinbach K Kursawe N CD8 MAIT cells infiltrate into the CNS and alterations in their blood frequencies correlate with IL-18 serum levels in multiple sclerosis. Eur J Immunol (2014) 44(10):3119–28. 10.1002/eji.201344160 25043505

[27] Maggi L Santarlasci V Capone M Peired A Frosali F Crome SQ CD161 is a marker of all human IL-17-producing T-cell subsets and is induced by RORC. Eur J Immunol (2010) 40(8):2174–81. 10.1002/eji.200940257 20486123

[28] Triebel F Hercend T. Subpopulations of human peripheral T gamma delta lymphocytes. Immunol Today (1989) 10(6):186–8. 10.1016/0167-5699(89)90321-6 2526644

[29] Hayday AC. [gamma][delta] cells: a right time and a right place for a conserved third way of protection. Annu Rev Immunol (2000) 18:975–1026. 10.1146/annurev.immunol.18.1.975 10837080

[30] Dieli F Poccia F Lipp M Sireci G Caccamo N Di Sano C Differentiation of effector/memory Vdelta2 T cells and migratory routes in lymph nodes or inflammatory sites. J Exp Med (2003) 198(3):391–7. 10.1084/jem.20030235 12900516 PMC2194087

[31] Kress E Hedges JF Jutila MA. Distinct gene expression in human Vdelta1 and Vdelta2 gammadelta T cells following non-TCR agonist stimulation. Mol Immunol (2006) 43(12):2002–11. 10.1016/j.molimm.2005.11.011 16423401

[32] Gibbons DL Haque SF Silberzahn T Hamilton K Langford C Ellis P Neonates harbour highly active gammadelta T cells with selective impairments in preterm infants. Eur J Immunol (2009) 39(7):1794–806. 10.1002/eji.200939222 19544311

[33] Polman CH O’Connor PW Havrdova E Hutchinson M Kappos L Miller DH A randomized, placebo-controlled trial of natalizumab for relapsing multiple sclerosis. N Engl J Med (2006) 354(9):899–910. 10.1056/NEJMoa044397 16510744

[34] Kivisakk P Mahad DJ Callahan MK Trebst C Tucky B Wei T Human cerebrospinal fluid central memory CD4+ T cells: evidence for trafficking through choroid plexus and meninges via P-selectin. Proc Natl Acad Sci U S A (2003) 100(14):8389–94. 10.1073/pnas.1433000100 12829791 PMC166239

[35] McFarland HF Martin R. Multiple sclerosis: a complicated picture of autoimmunity. Nat Immunol (2007) 8(9):913–9. 10.1038/ni1507 17712344

[36] Mastorodemos V Ioannou M Verginis P. Cell-based modulation of autoimmune responses in multiple sclerosis and experimental autoimmmune encephalomyelitis: therapeutic implications. Neuroimmunomodulation (2015) 22(3):181–95. 10.1159/000362370 24852748

[37] Olsson T. Cytokines in neuroinflammatory disease: role of myelin autoreactive T cell production of interferon-gamma. J Neuroimmunol (1992) 40(2–3):211–8. 10.1016/0165-5728(92)90135-8 1430152

[38] Panitch HS Hirsch RL Haley AS Johnson KP. Exacerbations of multiple sclerosis in patients treated with gamma interferon. Lancet (1987) 1(8538):893–5. 10.1016/S0140-6736(87)92863-7 2882294

[39] Jager A Dardalhon V Sobel RA Bettelli E Kuchroo VK. Th1, Th17, and Th9 effector cells induce experimental autoimmune encephalomyelitis with different pathological phenotypes. J Immunol (2009) 183(11):7169–77. 10.4049/jimmunol.0901906 19890056 PMC2921715

[40] Lowther DE Chong DL Ascough S Ettorre A Ingram RJ Boyton RJ Th1 not Th17 cells drive spontaneous MS-like disease despite a functional regulatory T cell response. Acta Neuropathol (2013) 126(4):501–15. 10.1007/s00401-013-1159-9 23934116

[41] Hirota K Duarte JH Veldhoen M Hornsby E Li Y Cua DJ Fate mapping of IL-17-producing T cells in inflammatory responses. Nat Immunol (2011) 12(3):255–63. 10.1038/ni.1993 21278737 PMC3040235

[42] Schirmer L Rothhammer V Hemmer B Korn T. Enriched CD161high CCR6+ γd T cells in the cerebrospinal fluid of patients with multiple sclerosis. JAMA Neurol (2013) 70(3):345–51. 10.1001/2013.jamaneurol.409 23599932

[43] Malmestrom C Haghighi S Rosengren L Andersen O Lycke J. Neurofilament light protein and glial fibrillary acidic protein as biological markers in MS. Neurology (2003) 61(12):1720–5. 10.1212/01.WNL.0000098880.19793.B6 14694036

[44] Housley WJ Pitt D Hafler DA Biomarkers in multiple sclerosis. Clin Immunol (2015) 161(1):51–8. 10.1016/j.clim.2015.06.015 26143623

[45] Kivisakk P Healy BC Viglietta V Quintana FJ Hootstein MA Weiner HL Natalizumab treatment is associated with peripheral sequestration of proinflammatory T cells. Neurology (2009) 72(22):1922–30. 10.1212/WNL.0b013e3181a8266f 19487650 PMC2690969

[46] Halstensen TS Scott H Brandtzaeg P. Intraepithelial T cells of the TcR gamma/delta+ CD8- and V delta 1/J delta 1+ phenotypes are increased in coeliac disease. Scand J Immunol (1989) 30(6):665–72. 10.1111/j.1365-3083.1989.tb02474.x 2481336

[47] Nilssen DE Muller F Oktedalen O Froland SS Fausa O Halstensen TS Intraepithelial gamma/delta T cells in duodenal mucosa are related to the immune state and survival time in AIDS. J Virol (1996) 70(6):3545–50. 8648688 10.1128/jvi.70.6.3545-3550.1996 PMC190229

[48] Fenoglio D Poggi A Catellani S Battaglia F Ferrera A Setti M Vdelta1 T lymphocytes producing IFN-gamma and IL-17 are expanded in HIV-1-infected patients and respond to *Candida albicans*. Blood (2009) 113(26):6611–8. 10.1182/blood-2009-01-198028 19395673

[49] Vermijlen D Brouwer M Donner C Liesnard C Tackoen M Van Rysselberge M Human cytomegalovirus elicits fetal gammadelta T cell responses in utero. J Exp Med (2010) 207(4):807–21. 10.1084/jem.20090348 20368575 PMC2856038

[50] Siegers GM Lamb LS Jr Cytotoxic and regulatory properties of circulating Vdelta1+ gammadelta T cells: a new player on the cell therapy field? Mol Ther (2014) 22(8):1416–22. 10.1038/mt.2014.104 24895997 PMC4435582

[51] Wu P Wu D Ni C Ye J Chen W Hu G gammadeltaT17 cells promote the accumulation and expansion of myeloid-derived suppressor cells in human colorectal cancer. Immunity (2014) 40(5):785–800. 10.1016/j.immuni.2014.03.013 24816404 PMC4716654

[52] Han A Newell EW Glanville J Fernandez-Becker N Khosla C Chien YH Dietary gluten triggers concomitant activation of CD4+ and CD8+ alphabeta T cells and gammadelta T cells in celiac disease. Proc Natl Acad Sci U S A (2013) 110(32):13073–8. 10.1073/pnas.1311861110 23878218 PMC3740842

[53] Miyake S Kim S Suda W Oshima K Nakamura M Matsuoka T Dysbiosis in the gut microbiota of patients with multiple sclerosis, with a striking depletion of species belonging to clostridia XIVa and IV clusters. PLoS One (2015) 10(9):e0137429. 10.1371/journal.pone.0137429 26367776 PMC4569432

[54] Ruff WE Kriegel MA. Autoimmune host-microbiota interactions at barrier sites and beyond. Trends Mol Med (2015) 21(4):233–44. 10.1016/j.molmed.2015.02.006 25771098 PMC5918312

[55] Chen J Chia N Kalari KR Yao JZ Novotna M Soldan MM Multiple sclerosis patients have a distinct gut microbiota compared to healthy controls. Sci Rep (2016) 6:28484. 10.1038/srep28484 27346372 PMC4921909

