# Supplementary material for: High Interferon-γ Uniquely in Vδ1 T Cells Correlates with Markers of Inflammation and Axonal Damage in Early Multiple Sclerosis
Source: Front Immunol. 2017 Mar 9;8:260. doi: 10.3389/fimmu.2017.00260 (PMC5343019; doi:10.3389/fimmu.2017.00260)
Supplement: Supplementary file 1 [file Image_1.PDF]

Supplementary Material

High interferon-γ uniquely in Vδ1 T cells correlates with neuronal damage in early multiple sclerosis

Avadhesh Kumar Singh, Lenka Novakova, Markus Axelsson, Clas Malmeström, Henrik Zetterberg, Jan Lycke, & Susanna L. Cardell*

*** Correspondence:** Susanna L. Cardell; E-mail: susanna.cardell@microbio.gu.se

Supplementary Figure 1

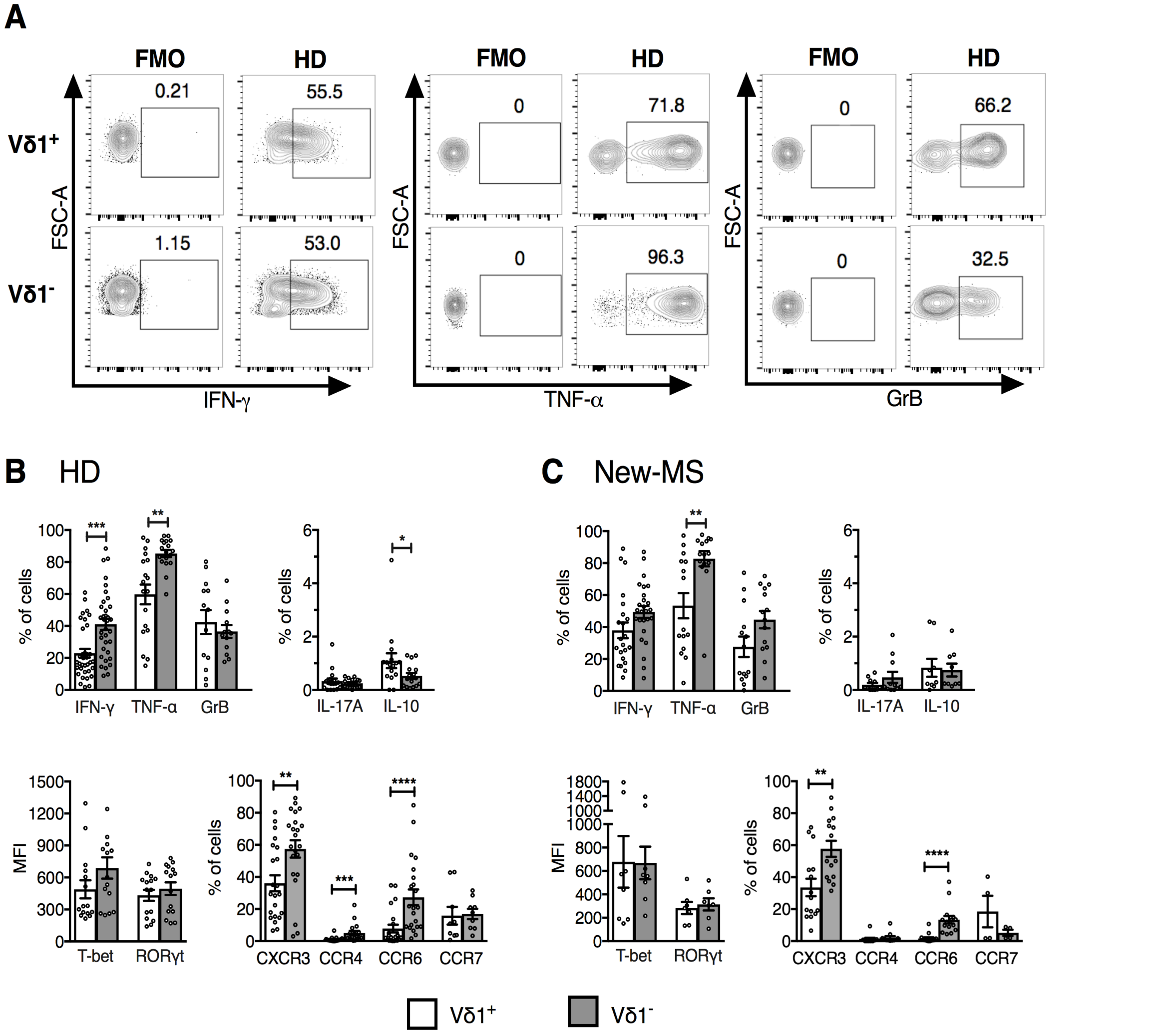

Figure S1 | Vδ1⁻ T cells had a more inflammatory phenotype than Vδ1 cells in HD. (A) Representative flow cytometric plots are shown for setting the gate for intracellular indicated cytokines. **(B)** Data from Figure 3 showing γδ T subsets from HD and New-MS, analyzed by flow cytometry for the expression of cytokines, transcription factors and chemokine receptors, are displayed for a direct comparison of Vδ1 and Vδ1⁻ T subsets. Bars represent mean ± SEM for Vδ1

and Vδ1^{-} T cells from HD (n = 35 for IFN-γ, n = 18 for TNF-α, n = 13 for GrB, n = 15 for T-bet and RORγt, n = 16 for IL-10 and IL-17A, n = 22 for CXCR3 and CCR6, n = 17 for CCR4 and n = 9 for CCR7) and New-MS patients (n = 21 for IFN-γ, n = 13 for TNF-α, n = 12 for GrB, n = 8 for T-bet and RORγt, n = 9 for IL-10 and IL-17A, n = 13 for CXCR3 and CCR6, n = 9 for CCR4 and n = 4 for CCR7). $^{*}P < 0.05$, $^{**}P < 0.01$, $^{***}P < 0.001$, $^{****}P < 0.0001$. Unpaired t-test with Welch's correction or Mann-Whitney *U*-test (two-tailed) was used to assess significance. FMO, fluorescence minus one; MFI, median fluorescence intensity.
